# Anticipatory Motor Planning and Control of Grasp in Children with Unilateral Spastic Cerebral Palsy

**DOI:** 10.3390/brainsci11091161

**Published:** 2021-08-31

**Authors:** Jennifer Gutterman, Trevor Lee-Miller, Kathleen M. Friel, Katherine Dimitropoulou, Andrew M. Gordon

**Affiliations:** 1 Department of Biobehavioral Sciences, Teachers College, Columbia University, New York, NY 10027, USA; jeg2211@tc.columbia.edu (J.G.); tl2517@tc.columbia.edu (T.L.-M.); 2 Burke Neurological Institute, Weill Cornell Medicine, White Plains, NY 10605, USA; kaf3001@med.cornell.edu; 3 Department of Rehabilitation & Regenerative Medicine (Occupational Therapy), Columbia University, New York, NY 10032, USA

**Keywords:** hemiplegia, motor planning, precision grip, isometric force scaling, fingertip force, object manipulation, prehension

## Abstract

Children with unilateral spastic cerebral palsy (USCP) have impairments in motor planning, impacting their ability to grasp objects. We examined the planning of digit position and force and the flexibility of the motor system in covarying these during object manipulation. Eleven children with a left hemisphere lesion (LHL), nine children with a right hemisphere lesion (RHL) and nine typically developing children (controls) participated in the study. Participants were instructed to use a precision grip with their dominant/less affected hand to lift and keep an object level, with either a left, centered or right center of mass (COM) location. Digit positions, forces, compensatory torque and object roll where measured. Although children with USCP generated a compensatory torque and modulated digit placement by lift-off, their index finger was either collinear or higher than the thumb, regardless of COM location, leading to larger rolls after lift-off especially for the RHL group. The findings suggest that while the kinetics of grasp control is intact, the kinematics of grasp control is impaired. This study adds to the understanding of the underlying mechanisms of anticipatory planning and control of grasp in children with USCP and may provide insights on how to improve hand function in children with USCP.

## 1. Introduction

Anticipatory control (motor planning) is an important component of grasping and manipulating objects. For example, to effectively drink from a cup, one needs to scale the size and orientation of their grasp to the cup before contact, as well as scale their fingertip forces to account for the cup’s texture, weight, and orientation to successfully reach the mouth. Skillful engagement in such tasks entails anticipatory control of movements at the level of kinematic (hand shaping and digit placement) and kinetic (digit force) control. Children and adolescents with unilateral spastic cerebral palsy (USCP) have significant problems in anticipatory planning of action that affect their efficiency in goal-directed functional tasks [1,2,3]. They do not take end-state comfort [4] into account when planning a multi-step action such as turning a doorknob [5] or placing a cylinder on a shelf [6]. Such impairments have been shown to occur even when using the less-affected hand [1,2], suggesting higher-level planning deficits [7].

Despite the global impairments reported above, other studies have suggested that impairments are lateralized primarily to the more-affected hand in children with USCP. When grasping and lifting, typically developing children (e.g., [8,9,10]) and adults (e.g., [11,12]) scale their fingertip forces prior to lift-off (before feedback becomes available) to object weight and texture based on prior experience with that object. This anticipatory force control prevents uncontrolled lifting accelerations or crushing of light objects, and exceedingly long lifting times or dropping of heavy objects. Although children with USCP are unable to anticipatorily scale their fingertip forces in their more-affected hand [13] unless they are provided extensive practice [14,15], they retain this ability in their less-affected hand (e.g., [16,17]). The discrepancy in global versus lateralized impairments may be due to the simplicity of the task (e.g., symmetrical mass distribution and using objects with a defined contact point). Alternatively, anticipatory control of kinematics and kinetics may be differentially impaired.

To date, studies of fingertip forces during object manipulation in USCP have constrained grasp locations to the site of small transducers located on the manipulated objects (e.g., [15,16,17]). Everyday object manipulation involves grasping of objects of uniform and non-uniform weight distributions, and a choice of where to place the fingertips (unconstrained grasp locations). When grasping objects with non-uniform weight distributions (e.g., a box with unequal distributed contents) without grasp location constraints, typically developed adults use prior experience to (1) place the digit on the heavier object side higher, and (2) use a larger vertical loading force in that digit during two-digit manipulation to achieve a compensatory torque preventing tilt [18,19,20,21]. On a trial-by-trial basis, subjects demonstrate a continuum of these two solutions [18]. Such flexibility highlights the increased complexity of this task compared to simply scaling digit forces to a uniform object weight with fixed contact points, and suggests the grasp is controlled using a higher (task) level planning process (i.e., motor equivalence [22]). It is unknown whether this more complex form of planning (involving anticipatory control of both kinematics and kinetics) would be affected globally (independent of hand) in children with USCP. Furthermore, such planning processes could be affected by the side of the brain the lesion is located. While some studies have shown greater impairment in action selection for participants with left hemisphere lesions [23,24], others have suggested that action planning is not lateralized [25,26]. The effects of lesion location on grasp kinetics in children with USCP has not been studied to date.

The present study is the first to simultaneously examine both the planning of digit positions and forces in the less-affected hand of children with USCP. We hypothesized that children with USCP, in particular those with left hemisphere lesions, will show a reduced ability to prevent object roll compared to typically developing children, and less flexibility of the motor system in covarying digit positions and forces.

## 2. Materials and Methods

Participants: Eleven children with USCP with a left hemisphere lesion (LHL group: 4 females, age (M ± SD) = 12.45 ± 2.30 years), nine children with USCP with a right hemisphere lesion (RHL group: 7 females, age (M ± SD) = 11.33 ± 2.35 years) and nine typically developing right-handed children (control group: 7 females, age (M ± SD) = 11.29 ± 2.56 years) were included in the study (See Table 1 for participant characteristics). Lesion location was determined from structural Magnetic Resonance Imaging (MRI) scans without sedation prior to participation. MRI scans were performed on a Siemens Prisma MRI Scanner in the Citigroup Biomedical Imaging Center (165 slices at a resolution of 256 × 256 px). The children with USCP were a sample of convenience, participating in summer upper extremity rehabilitation camps from 2017–2019 at Teachers College, Columbia University. The inclusion criteria for the rehabilitation camps included (1) diagnosis of USCP, (2) mainstreamed in an age-appropriate classroom, (3) ability to follow instructions while lifting an object during the screening process, (4) ability to lift the more-affected arm 15 cm above a table surface and grasp light objects and (5) seizure-free after age 2 without anti-seizure medication. Exclusion criteria included (1) health problems unassociated with USCP, (2) uncontrolled seizures, (3) uncorrected visual impairments, (4) orthopedic surgery on the more-affected hand within 1 year before camp, (5) non-removable metallic objects in the body and (6) botulinum toxin in the more-affected upper extremity within 6 months of participating. The control group included typically developing right-handed children mainstreamed in schools. Informed assent/consent was obtained from participants and their legal guardians. Eight children with USCP who did qualify for the rehabilitation camps were excluded from this study due to non-compliance with instructions.

**Figure 1:**
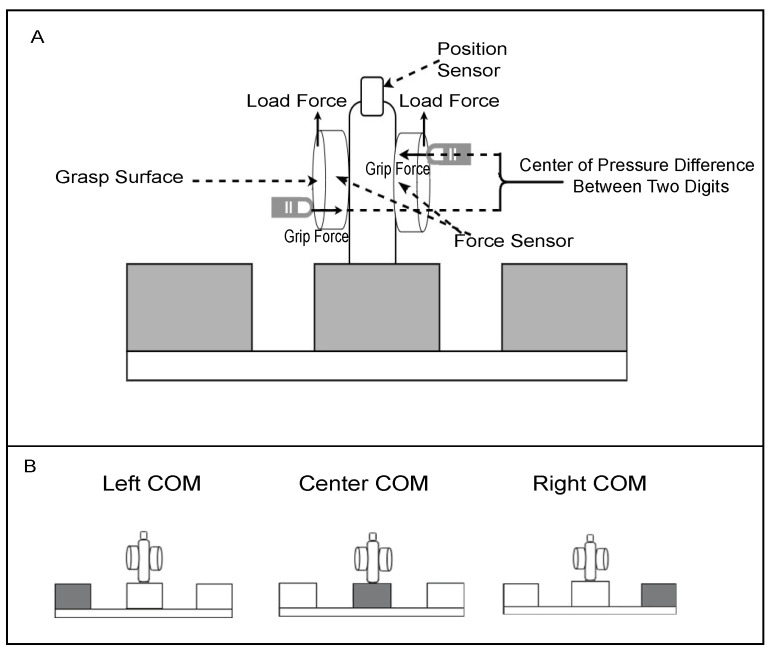
(**A**) custom-made device with force transducers and position sensor. (**B**) Shaded box represents where the heavy weight (180 g) is for each center of mass (COM) location.

Apparatus: A custom-made inverted-T shaped object (length 17 cm, height 13.3 cm) was used (Figure 1). There were two multi-axis force transducers (Mini 40 Force/Torque transducer; ATI Industrial Automation) situated on the grip surfaces measuring grip force, load force and torques (400 Hz, resolution = 0.01 N, 0.01 N, 0.125 Nmm, respectively). The grip surfaces were covered with 100-grit sandpaper. An electromagnetic sensor (Polhemus Fastrak, 120 Hz, 0.005 mm of range, and 0.025° resolution) was mounted on top of the device in order to measure the position and roll of the object. A weight of 180 g was placed in the left, center or right of the object, shifting the object’s center of mass (COM) 3 cm from the object’s center during the left and right COM conditions, while keeping the appearance the same (Figure 1B). The total weight of the device with the weights was 512 g, resulting in an external torque of ±16 Ncm of the object.

Task/Procedures: For this study, children used their dominant/less-affected hand. The less-affected hand of children with USCP was chosen because (1) their more-affected hand was often too weak, exhibited wrist range of motion limitations and was postured into flexion and pronation, limiting use for this task, and (2) we were interested in “higher level” motor planning processes that are independent of impairments of the affected hand. Children sat with their hands on their lap at an adjustable table and were instructed to take their less-affected/dominant hand off their lap and lift the object using a precision grip (thumb and index finger) 5 cm (indicated with a vertical marker) off the support surface at the first auditory cue. The object was held in that position until they heard an auditory cue 5 s later, when they would place the object back on the table and release it. Children were instructed that the goal of the task was to keep the object as level as possible. The task was performed on the object in the left, center and right COM conditions. For each of these conditions (order counterbalanced across participants), participants performed five practice trials followed by 10 blocked test trials, for a total of 45 lifts. The practice and test trials were analyzed separately. There was approximately 10 s between each lift, and a rest of 3 min was provided as the mass location was changed out of view of the subject.

Data Processing: Throughout the lifts, grip force, load force and torques applied to the grip surfaces were recorded by the two force transducers using custom written software in SC/Zoom (Umeå University, Sweden). In order to examine anticipatory planning of digit forces and position, grip force, load force, center of pressure and torque were analyzed at lift onset before any feedback signaling the COM location became available. Lift onset was defined as the point at which the vertical position of the object exceeded a value of 2 mm off the table surface and continued thereafter.

Main Outcomes:

1. Peak object roll—recorded within 500 ms after lift onset (lift-off), the angle of the object in the frontal plane. Positive values for peak object roll signify a counterclockwise roll (towards the left) and negative values signify a clockwise roll (towards the right).
  - Peak velocity of roll = peak object speed and direction of the rotation of the object during the initial roll of the object.
  - Static roll = the degree the initial roll had been corrected at mid-lift.
2. Load force (LF)—vertical component of the force counteracting weight and inertia produced by each digit at lift-off.
  - Load force difference (LF Difference) = LF left − LF right. Positive values indicate larger LF from the digit on the left grip surface, whereas negative values indicate larger LF from the digit on the right grip surface.
3. Grip Force (GF)—average normal (perpendicular to the grip surface) component of the force produced by each digit at lift-off.
4. Center of pressure (COP)—vertical coordinate of the point of application of the digit on the grip surface, measured in (cm) at lift-off, signaling digit position.
  - Center of pressure difference (COP Difference) = COP left − COP right. Positive values indicate higher left digit than right digit COP, whereas negative values indicate higher right digit than left digit COP.
5. Compensatory torque (Tcom)—anticipatory torque generated by the digits, in response to object torque, at lift-off. A positive Tcom signifies a clockwise torque, while a negative Tcom signifies a counter-clockwise torque.
  $$\mathrm{Tcom}=\mathrm{LF}\;\mathrm{Difference}\;\times \;\frac{1}{2}(\mathrm{grip}\;\mathrm{width})+(\mathrm{GF}\;\times \;\mathrm{COP}\;\mathrm{Difference})$$
  - Where half of grip width was 2.15 cm.
  - Object torque was ±16 Ncm.

Statistical Analysis: To determine when performance stabilized during practice, we ran a mixed ANOVA on the five practice trials for each COM condition on object roll. An additional t-test was run between the first and last experimental trials to confirm the stability of performance. Furthermore, we ran one-way repeated measures ANOVAs between conditions for each group, to examine condition order effects, if any. A 3 (group: TD controls, LHL, RHL) × 3 (COM: left, center, right) ANOVA with repeats on the latter were performed on all variables. Greenhouse–Geisser correction was used when the assumption of sphericity was not met. The Shapiro–Wilk normality test was run, and the assumption of normality met for all variables. The homogeneity of variance assumption was examined using Levene’s Test. This assumption was not met for Tcom and GF. Therefore, non-parametric statistical analyses were used on these variables (Kruskal–Wallis Test with post-hoc Mann–Whitney U for between group analysis and Friedman test with post-hoc Wilcoxin signed rank tests for within group analysis). Significance was considered at the *p* < 0.05 level. For all post-hoc statistical comparisons, we applied a Bonferroni correction. Spearman rank correlations were performed to study the association between peak roll and Tcom for each COM location, for each group. Pearson correlations were performed to examine the association between LF difference and COP difference for each COM location and group. Fischer’s r-z transformation was used to calculate the mean correlations. Statistical analyses were performed using SPSS software (SPSS 27.0, Chicago, IL, USA).

## 3. Results

**Figure A1:**
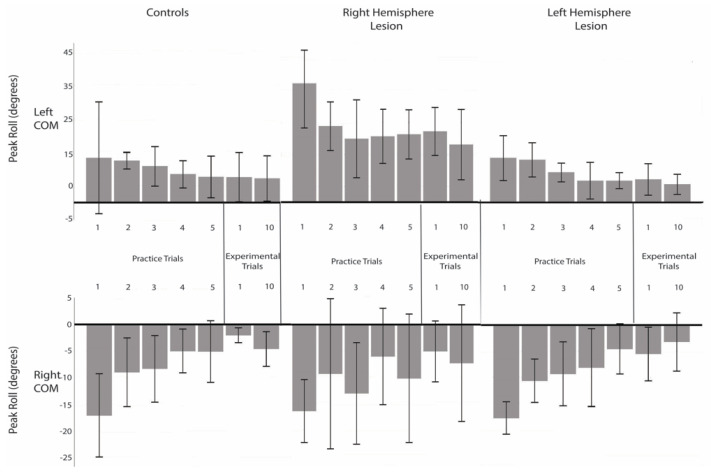
Mean peak object roll for each group for the left and right center of mass (COM) locations for the practice trials and the first and last experimental trials.

During the first practice trial, peak roll was large when the COM was located on the left and right side of the object during practice (left COM main effect of trial, (*F*(2.05, 35.11) = 8.34, *p* < 0.01); right COM main effect of trial (*F*(4,12) = 11.95, *p* < 0.001). However, post-hoc analysis of the mixed ANOVA revealed that the groups familiarized themselves with the object by the last practice trial, as indicated by the lack of statistically significant differences between these trials (trials 3–4, 3–5, 4–5 for left COM; 2–3, 2–5, 3–4, 4–5 for right COM) (See Appendix A Figure A1). In addition, the results demonstrated that performance was stable between the first and last experimental trials for each group (See Appendix A Figure A1). Therefore, the mean/median of the 10 experimental trials were used for further analysis. There were no order effects of COM (*p* > 0.05 in all cases).

**Figure 2:**
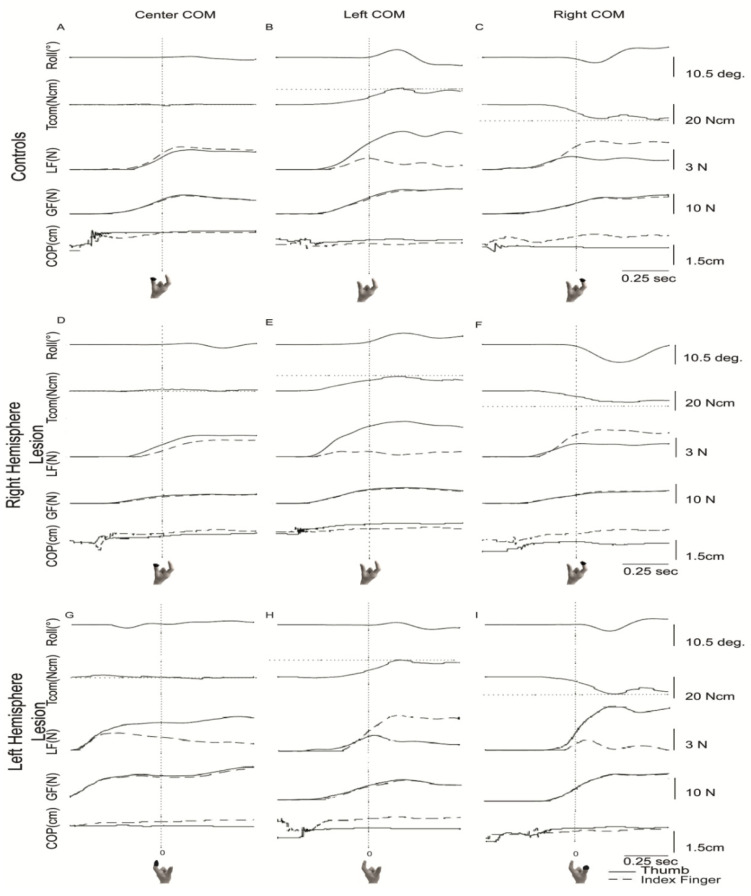
Representative trials for left (**B**,**E**,**H**), center (**A**,**D**,**G**) and right (**C**,**F**,**I**) center of mass locations. Vertical dotted lines indicate lift onset. Horizontal dotted lines for Tcom represent target Tcom. For LF, GF and COP, solid lines represent the thumb, whereas the dashed lines represent the index finger. The black circle on the picture of the hand below the graph represents which digit was on the side the heavy COM was located. Tcom = compensatory torque; COP = center of pressure; LF = Load Force, GF = grip force.

Subsequent analyses of the test trials (trials 6–15) reveal further information about how roll was reduced. Figure 2 shows traces of roll, Tcom, LF, GF and COP for one test trial of a representative participant from each group when the COM was located on the center, left, and right side of the object. As shown in Figure 2A, the control participant had near zero roll and Tcom, minimal LF differences between digits and collinear digit placement on the object when the COM was located in the object’s center. When the COM location was on the left (Figure 2B) and right (Figure 2C) side of the object, a small roll in the direction of the COM location (i.e., roll toward the heavier side in each condition) occurred. Although Tcom was not fully developed until after lift-off, a substantial amount of torque occurred before lift-off (vertical line at time zero), indicating anticipation of the COM location (Figure 2B,C). The torques were achieved by a larger LF and higher COP on the COM side (i.e., thumb higher when the COM location was on the left, index finger higher when the COM location was on the right), GF development that was similar between digits and continued to increase after lift-off and higher COP on the COM side. The participant in the RHL group, similar to the control participant, had slight roll after lift-off, with Tcom nearly zero (Figure 2D) when the COM was in the center. However, the RHL participant had larger left digit LF, with a near collinear digit placement. When the COM location was on the left (Figure 2E) and right (Figure 2F), there was roll in the direction of the COM, and similar Tcom and LF generation as the control participant. However, when the COM was on the left, GF increased only slightly after lift-off, and digit placement was collinear. When the COM location was on the right (Figure 2F), although Tcom was generated in the appropriate direction, target Tcom was not achieved, even after lift onset. Still, the forces and digit placement were partitioned similarly to the control participant. For the participant in the LHL group, Tcom and peak roll were similar to the control participant (Figure 2G–I). LF production and digit placement differed when the COM was centered, as digit placement and LF production were not collinear (Figure 2G). Although the development of forces and digit placement were similar to the control participant when the COM was on the left (Figure 2H), when the COM was on the right (Figure 2I), digit placement differed (collinear digit placement).

### 3.1. Peak Object Roll and Compensatory Torque

**Figure 3:**
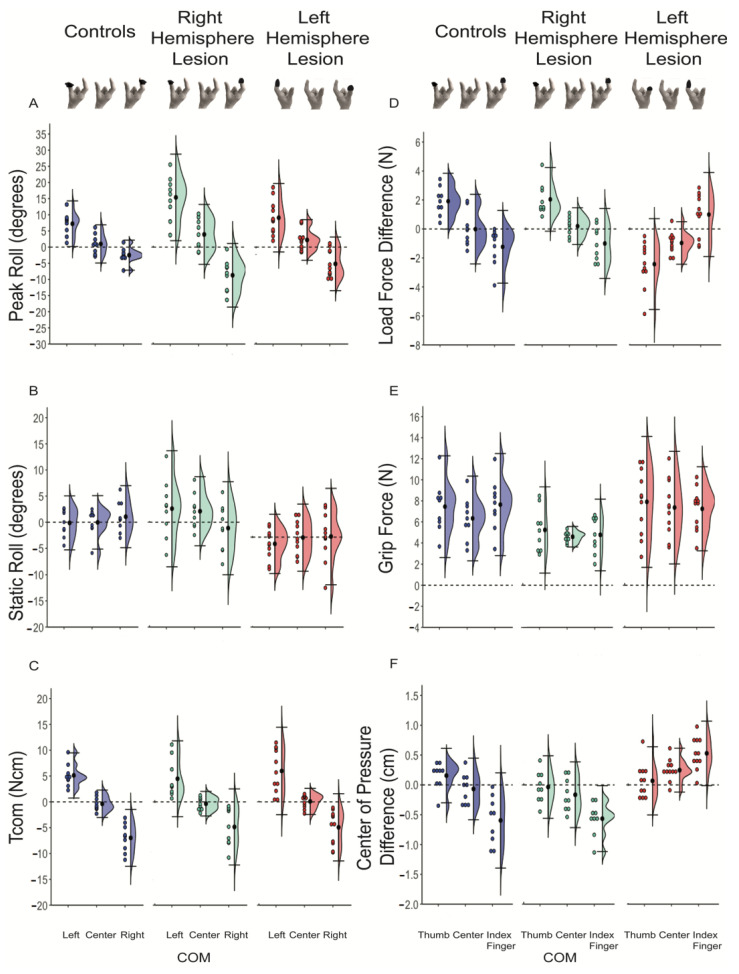
Violin plots (**A**–**F**). Group means for each group for the left center and right center of mass locations are denoted by the black dot, with ±standard error (SE) for peak roll, static roll and Tcom. Group means for each group for the thumb, center and index finger COM locations are denoted by a black dot, with ± SE for load force difference, grip force and center of pressure difference. Blue graphs represent the control participants, green represents the participants in the right hemisphere lesion group and red represents the participants in the left hemisphere lesion group. The black circle on the picture of the hand below the graph represents which digit was on the side the heavy COM was located. (**A**) Peak roll, (**B**) compensatory torque (Tcom), (**C**) load force difference (LF difference), (**D**) grip force (GF), (**E**) Center of Pressure Difference (COP difference), (**F**) static roll.

Figure 3 and Table 2 show the above findings were representative of the subjects we tested. There was typically an object roll in the direction of COM location when the COM was on the left or right side of the object, of which the extent differed between groups (significant group X COM interaction, *p* < 0.05) (See Figure 3A). Post-hoc analysis showed when the COM was located on the left side of the object, the LHL and control groups had a smaller peak object roll in comparison to the RHL group (*p <* 0.016). When the COM was located on the right side, the control group corrected the initial clockwise roll of the object toward the horizontal 5.72° more than the RHL group (*p* < 0.016). Furthermore, the RHL group showed a reduced ability to minimize static roll for all conditions during mid-lift (Figure 3B) (significant group X COM interaction, *p* < 0.05).

Friedman test results showed that all three groups demonstrated a generation of Tcom in the appropriate direction (Figure 3C; Table 2) (all *p* < 0.05). Although none of the groups achieved target Tcom (±16 Ncm) at lift-onset, post-hoc comparisons revealed that Tcom differed and was applied in the appropriate direction when the COM was located on the left (clockwise torque) and right (counterclockwise torque), and around zero for the center COM location (all *p* < 0.016 except RHL between center and right COM locations). There were no significant differences in the magnitude of Tcom between groups.

### 3.2. Components of Compensatory Torque

All groups modulated LF difference based on the COM location (significant main effect) (Figure 3D; Table 2). Post-hoc tests demonstrated that on average, when the COM was located on the left, the LF of the thumb for the control and RHL groups and the index finger for the LHL group (left digit LF), was larger than the index finger for the control and RHL groups, and the thumb for LHL group (right digit LF) (*p* < 0.016). When the COM was centered, LF was similar between digits. Post-hoc analysis further showed that for the right COM, the index finger for the control and RHL groups, and the thumb for LHL group (right COM location) was larger than the thumb for the control and RHL groups, and the index finger for the LHL group (left digit LF) (*p* < 0.016). The LHL group had a smaller LF difference when the COM was on the side of the left digit and a more negative LF difference when the COM was on the center and on the side of the right digit compared to the other two groups (significant main effect of group, *p* < 0.05).

The Kruskal–Wallis test demonstrated that the GF was significantly different between groups when the COM was located on the right side (index finger for RHL and control group and thumb for the LHL group) (*p* < 0.05). Post-hoc analysis revealed the GF of the control group was larger than the GF in the RHL group when the COM was on the side of the thumb for these groups (*p* < 0.016) (Figure 3E).

All three groups modulated digit placement based on the COM, as measured by COP difference (significant main effect of COM, *p* < 0.05). Post-hoc analysis showed that while all groups modulated COP difference depending on the COM location (*p* < 0.016), groups maintained this differently (significant main effect of group, *p* < 0.05). Post-hoc analysis revealed significant differences between the LHL group and the RHL and control groups (*p* < 0.016). As shown in Figure 3F and Table 2, the COP difference across all COMs for the LHL group (all left hand dominant) were all positive, with the index finger placed higher or collinear to the thumb. The COP difference for the RHL group were negative, with the index finger placed higher or collinear than the thumb. For the control group, the digit that was placed higher depended on where the COM was, with a positive COP difference when the COM was on the side of the thumb (left digit COP), negative COP difference when the COM was on the side of the index finger (right digit COP) and collinear when the COM was in the center.

**Figure 4:**
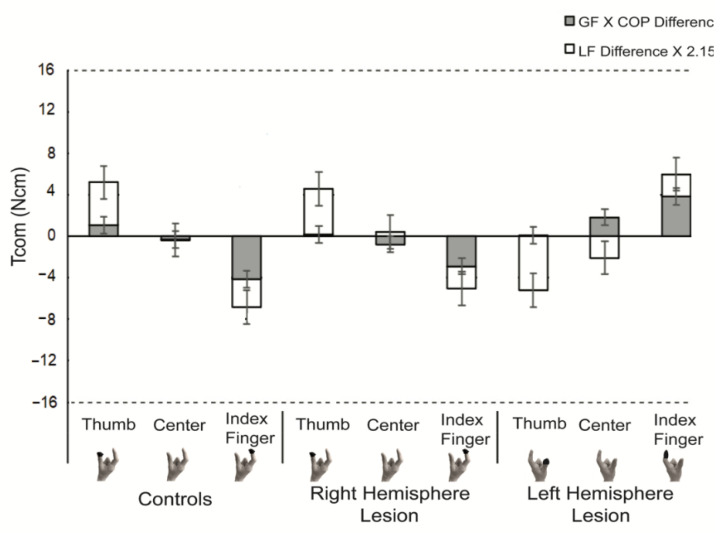
Stacked bar graph of Tcom components. Tcom = compensatory torque; LF = load force; GF = grip force; COP = center of pressure. The dotted horizontal line represents target Tcom. The black circle on the picture of the hand below the graph represents which digit was on the side the heavy COM was located.

Since Tcom opposing external torque of the object is the result of varying several parameters, Figure 4 demonstrates the contribution of each of these parameters for each group and COM. As discussed above, LF difference was modulated based on the COM, but the magnitude varied between groups. The other component of torque (GF X COP difference) was also modulated based on the COM (significant main effect of COM, *p* < 0.05). This component of torque on average contributed more to the generation of clockwise torque in the LHL compared to the RHL and control groups (significant main effect of group, *p* < 0.05).

### 3.3. Relationship and Discrepancy between Object Roll and Tcom

Although Tcom was generated to a statistically similar extent between groups, peak roll was not minimized to a similar degree between groups, as noted above. Although the RHL group had the smallest correlation between Tcom and peak roll (Table 3) (TD controls: *r =* −0.67, Left LHL: *r* = −0.60, RHL: *r* = −0.52), the differences of the correlations were not significant.

Due to the discrepancy between Tcom and peak roll, and the moderate correlations between these variables, a 3 group (TD controls, LHL, RHL) × 3 COM (left, center, right) ANOVA with repeats on the latter were performed on the velocity of the roll and the time to peak roll. The RHL group had a higher peak velocity of the roll in comparison to the control group when the COM was on the right side of the object (significant group × COM interaction, *p* < 0.05). There were no differences in the time it took to correct for the roll between groups.

### 3.4. Covariation of Digit Forces and Placement

**Figure 5:**
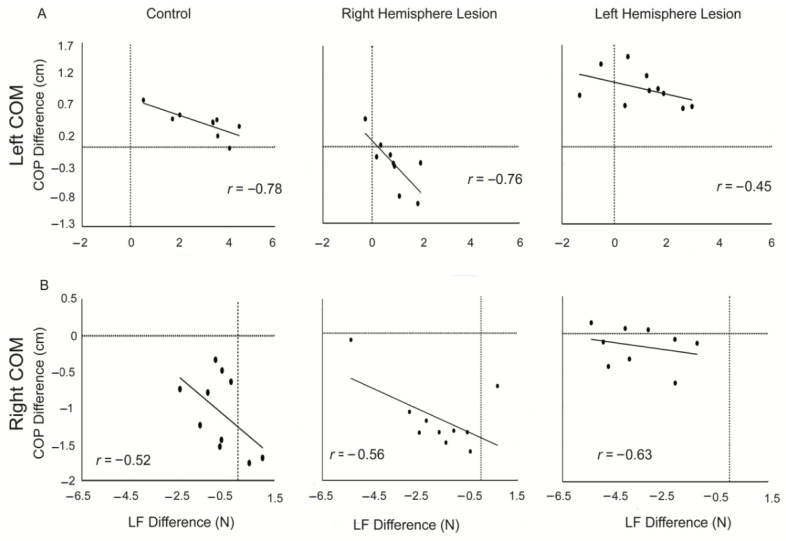
Representative scatter plot of the covariation of load force (LF) difference and center of pressure (COP) difference for each individual trail for a participant from each group for the (**A**) left and (**B**) right center of mass (COM) locations. Pearson’s r correlation coefficient is shown for the 10 experimental trials.

All groups showed a moderate to high correlation between LF difference and COP difference (controls: *r* = −0.79, RHL: *r* = −0.65, LHL: *r* = −0.70), suggesting covariation of these variables (See Table 3). There were no significant differences between groups. Figure 5 demonstrates representative plots of the covariation of digit forces and placement across the 10 test trials from a participant in each group for when the COM was on the left and right side of the object. For the participants in the control and LHL groups, when the COM was on the left side of the object, the COP difference and LF difference was mostly positive (Figure 5A). The COP difference was offset by the LF difference (i.e., the smaller the COP difference, the greater the LF difference) for the control and LHL groups. However, while the RHL group had a moderate correlation between these two variables, the COP difference was negative (digit on the side of the object opposing the COM was placed higher), with a more positive LF difference (larger LF of the digit on the left side of the object (thumb) compared to the digit on the right side of the object (index finger) to compensate. When the COM was on the right (heavier) side of the object, the LHL group had a moderate, negative correlation (Figure 5B). However, the difference in digit placement was all near zero, indicating near collinear digit placement. This was offset by the large negative LF difference. The control and RHL groups (Figure 5B) demonstrated that although the correlation was slightly lower, the smaller the LF difference, the greater the COP difference.

## 4. Discussion

The present study is the first to examine the flexibility of the motor system in covarying digit position and forces during object manipulation, and whether anticipatory control of kinematics and kinetics is differentially impaired in children with USCP. Children with USCP were able to modulate digit placement and forces in their less-affected hand. However, they used a maladaptive digit placement strategy when the COM was located on the same side as the thumb, leaving their thumb below or collinear to the index finger. This led to the USCP groups, in particular the RHL group, having larger rolls after lift-off. The findings suggest that while the kinetics of grasp control are largely intact, the kinematics of grasp control are impaired. These findings and their implications are discussed below.

### 4.1. Children with USCP Have Anticipatory Control of Digit Forces and Torque

To successfully minimize roll of an object with an asymmetric mass distribution, subjects need to develop torque to counter the external torque of the object by modulating digit positions and/or forces. In contrast to our hypothesis, all groups were shown to have the ability to scale load forces, with higher load forces on the digit located on the heavier side, in order to develop compensatory torque based on prior experience with the object. However, even after performance stabilized, target compensatory torque was not reached at lift onset when the COM was on the left or right. Thus, additional torque was applied after lift-off to further minimize roll. The anticipatory control of forces and torque is in line with prior studies that demonstrated children with USCP generally have intact anticipatory control of kinetics in the less-affected hand [7,16,17,27].

### 4.2. Children with USCP Demonstrated a Maladaptive Digit Placement Strategy

Although children with USCP did generate compensatory torque and modulate digit placement (measured by COP) by lift-off, their index finger was either collinear or higher than the thumb, regardless of COM location. In contrast, the control group placed the digit on the COM side higher, thereby contributing to Tcom generation. Due to the near collinear digit placement for the RHL and LHL groups (when the COM was on the side of thumb) (Figure 4), the LF difference between digits was a larger component of Tcom generation.

Since additional torque after lift-off was required, the digit placement used where the thumb is collinear or below the index finger prevents the ability to create a passive torque simply by creating a lever arm, and thus the children with USCP rely on further (active) partitioning of digit forces after lift-off to counteract object roll. The larger LF differences that were necessary for the more active torque required more energy than the passive contribution of digit position [18].

The biomechanical disadvantage of not using the passive lever arm in order to account for subsequent actions is in line with past studies that demonstrated impairments relating to kinematic scaling and end-state comfort in the less-affected hand [1,2,7,16,17]. This is comparable to previous studies where a multi-step action was performed such as placing a cylinder on a shelf [6], where children with USCP are able to grasp the object, but do not account for what will be done with the object (i.e., place a cylinder on a shelf, or in this study to keep the object level). It has also been shown that digit placement is learned faster than digit forces [20], and that digit placement may be explicit knowledge, while partitioning of forces may be implicit knowledge [28]. It has been recommended that children with USCP focus on implicit learning rather than explicit learning to enhance motor skills [29,30]. Therefore, the differentiation between digit forces and placement may be due to an effect of explicit knowledge of object properties of digit placement. Furthermore, it is conceivable that children with USCP position their thumb during precision grip as healthy adults do during other grasp patterns (e.g., five-digit grasp), with the thumb slightly lower than the index finger in all COM locations [31,32].

### 4.3. Lesion Side Influenced the Ability to Minimize Peak Roll

There was a larger peak roll for both lesion USCP groups, although this was only significant for the RHL group. In addition to the larger peak roll and higher velocity of roll for the RHL group, there was a reduced correction of this error when holding the object in the static phase during the left and center COM locations (See Figure 2). The RHL did not respond as robustly to the roll, even though they corrected roll as quickly as the LHL and control groups. In contrast to our hypothesis that those with LHLs would have larger rolls after lift-off than children with RHLs, those with RHLs had a significantly larger roll. This could be attributed to the specialization of the right hemisphere in accurately achieving a goal end position when there is an adaptation in the environment and correcting ongoing actions or movements, as shown in adults with strokes [33]. Therefore, a lesion affecting the right hemisphere could affect the ongoing actions after lift-off, leading to a larger roll that is not corrected as much. Additionally, the lack of correction to the roll could relate to greater hemineglect deficits [34] or allocentric visuospatial deficits in children with RHLs [35]. Furthermore, the lower GF trend in the RHL group, combined with the maladaptive digit placement, may have made it more challenging to apply torque to correct roll after lift-off.

### 4.4. Covariation of Digit Placement and Forces in Order to Achieve Tcom

In healthy adults, compensatory torques are achieved with a combination of force scaling and digit positioning along a spectrum (e.g., [18,31,36]; i.e., they use motor equivalence [22]). Given this flexibility, on trials where the digits are more collinear, the LFs are partitioned during lift-off to a greater extent. Similarly, on trials where there are larger partitions of digit placement, the LFs are partitioned to a lesser extent. Despite the reduced magnitude of scaling of digit positions in the USCP groups when the COM was on the side of the thumb, they showed similar covariation of digit placement and forces to controls, which did not support our hypothesis. However, the covariation between digit placement and forces was less compared to healthy adults (typically 0.8 to 0.9) [18,31,36], likely reflecting continued development of grasp coordination until adolescence [8]. Alternatively, it could be due to the considerably lower external torque of the object compared to ones used in adult studies (e.g., [20,21]).

### 4.5. Limitations

One limitation is the small sample size when lesion side is considered. Furthermore, while there were children with USCP whose left hand was less affected than the right (LHL group), there were not left-handed control participants or control participants who used their left hand in this study, and thus the use of a left hand may have influenced our findings for this group. However, the strategy of keeping the index finger higher or collinear to the thumb was similar for both LHL and RHL participants. The amount of roll after lift-off was significantly different between USCP groups though, and therefore hemispheric differences should be explored further. Since there was a lack of consequence for poor performance, the attention span and motivation of the children could have influenced their performance, thus possibly explaining their reduced corrective actions after roll. Additionally, there could be possible gender effects in the control group, as there were more females than males in this group. Furthermore, we did not use a wide range of external torques for this experiment, and therefore do not know if the same strategies would be used if the external torque of the object varied. Finally, we examined the less-affected hand in children with unilateral CP, as the focus was on “higher-level” planning independent of the effector. Because of biomechanical limitations, we did not examine the more-affected hand, and thus we do not know the extent to which the findings may explain manual deficits in this hand.

## 5. Conclusions

Although children with USCP do maintain motor equivalence and modulate digit forces and position in order to generate the appropriate torque at lift-off, the placement of the thumb when the COM was located on that side resulted in a disadvantageous position. This strategy, in particular for those with right hemisphere lesions, may not be biomechanically optimal to reduce tilt of an object after it lifts off from the support surface. While kinematics and kinetics have been studied in isolation, through the use of this task, it was possible to examine planning of digit positions and forces simultaneously. This study adds to the understanding of the underlying mechanisms of anticipatory planning and control of grasp in children with USCP and may provide insights to provide more individualized interventions to help improve hand function in children with USCP.

## Figures and Tables

**Table 1:** Participant information.

Baseline Demographic and Clinical Characteristics	Children with USCP (Left Hemisphere Lesion) (*n* = 11)	Children with USCP (Right Hemisphere Lesion) (*n* = 9)	Control Group (*n* = 9)
Age, years	12.45 ± 2.30	11.33 ± 2.35	11.29 ± 2.56
Gender, females	4 (36.4%)	7 (77.8%)	7 (77.8%)
MACS Score			
I	4 (36.4%)	3 (33.3%)	N/A
II	4 (36.4%)	6 (54.5%)	N/A
III	3 (27.3%)	0 (0%)	N/A
Jebsen Score	41 ± 67.45	43.97 ± 298.30	N/A

Note: Age in years and months; values are means ± SD or %; MACS = manual ability classification system; Jebsen = Jebsen-Taylor Test of Hand Function, where lower time mean is better performance. One Jebsen score for the RHL group was not collected and therefore not included in the Jebsen score mean.

**Table 2:** Mean and standard deviations of each variable for each group for each center of mass. *p* values for the interaction, group and center of mass main effects. Median, interquartile range, chi squared and *p* values for non-parametric variables (Tcom and GF). Tcom = compensatory torque; LF diff = load force difference; GF = grip force; COP diff = center of pressure difference; C = controls, RHL = right hemisphere lesion; LHL = left hemisphere lesion; LCM = left center of mass, CCM = centered center of mass, RCM = right center of mass.

Main Variables	Left COM			Center COM			Right COM			Group Effect F, *p* Value (η_p_^2^)	COM Effect *F*, *p* Value (η_p_^2^)	Interaction *F*, *p* Value (η_p_^2^)
	C	RHL	LHL	C	RHL	LHL	C	RHL	LHL			
Peak Roll (degrees)	7.24 ± 3.54	15.38 ± 6.70	9.09 ± 5.30	0.98 ± 2.96	3.93 ± 4.63	2.22 ± 3.14	−2.99 ± 2.59	−8.71 ± 4.92	−5.18 ± 4.15	–	–	*F*(2.78,36.18) *=* 6.03, *p* < 0.01(0.32)
Tcom (Ncm)	4.31 (3.82, 5.70)	3.13 (1.80, 7.95)	5.34 (2.69, 11.00)	−0.98 (−1.36, 0.65)	−0.39 (−1.56, 0.53)	−0.71 (−1.40, 1.09)	−6.53 (−10.00, −4.38)	−4.12 (−8.28, −1.02)	−3.91 (−7.40, −1.68)	**LCM:** *p* = 0.594 **CCM:** *p* = 0.903 **RCM:** *p* = 0.168	**C:** χ^2^ (2) = 18.00, *p* < 0.001 **RHL:** χ^2^ (2) = 16.22, *p* < 0.001 **LHL:** χ^2^ (2) =20.18, *p* < 0.001	–
LF Diff (N)	1.91 ± 0.96	2.04 ± 1.10	1.00 ± 1.45	−0.01 ± 1.20	0.19 ± 0.63	−1.00 ± 0.73	−1.23 ± 1.25	−1.00 ± 1.20	−2.43 ± 1.57	*F*(2,26) =7.72, *p* < 0.01 (0.37)	*F*(1.38, 35.94) = 61.51, *p* < 0.001 (0.70)	*F*(2.77,35.95) = 0.09, *p* = 0.97 (0.01)
GF (N)	7.62 (5.76, 8.37)	4.99 (3.59, 7.20)	7.89 (5.89, 8.23)	6.20 (4.72, 7.77)	5.09 (4.29, 5.23)	7.29 (4.87, 8.81)	8.09 (6.40, 9.12)	4.69 (3.05, 6.48)	8.53 (4.83, 11.11)	**LCM:** *p* = 0.14 **CCM:** *p* = 0.06 **RCM:** *p* = 0.02	**C:** χ^2^ (2) = 6.89, *p* = 0.03 **RHL:** χ^2^ (2) = 0.22, *p* = 0.90 **LHL:** χ^2^ (2) = 2.18, *p* = 0.34	–
COP Diff (cm)	0.16 ± 0.23	−0.04 ± 0.26	0.53 ± 0.27	−0.07 ± 0.26	−0.17 ± 0.28	0.25 ± 0.18	−0.60 ± 0.40	−0.57 ± 0.28	0.07 ± 0.29	*F*(2, 26) = 22.15, *p* < 0.001 (0.63)	*F*(2,25) = 31.37, *p* < 0.001 (0.72)	*F*(4,50) = 2.02, *p* = 0.106 (0.14)
Peak Velocity of Roll	51.73 ± 18.64	86.90 ± 39.82	63.46 ± 31.61	8.25 ± 24.71	30.05 ± 29.40	22.97 ± 22.40	−24.13 ± 14.82	−60.16 ± 30.32	−39.15 ± 32.84	–	–	(*F*(2.90, 37.69) = 3.64, *p* = 0.02 (0.22)
Static Roll	−0.13 ± 2.59	2.60 ± 5.54	−1.12 ± 2.47	−0.03 ± 2.56	2.11 ± 3.30	−0.06 ± 2.80	1.06 ± 3.00	−1.12 ± 4.43	0.11 ± 4.02	–	–	*F*(3.00,38.91) = 2.89, *p* < 0.05 (0.18)
Time to Peak Roll	0.24 ± 0.07	0.27 ± 0.07	0.18 ± 0.08	0.18 ± 0.04	0.20 ± 0.04	0.18 ± 0.05	0.23 ± 0.07	0.46 ± 0.71	0.22 ± 0.06	*F*(2,26) = 1.82, *p* > 0.18 (0.12)	*F*(1.04, 26.93) = 2.02, *p* = 0.17 (0.07)	*F*(2.07, 26.93) = 0.83, *p* = 0.45 (0.06)
COP diff X GF	1.07 ± 1.81	0.18 ± 1.61	3.85 ± 2.13	−0.33 ± 1.52	−0.78 ± 1.30	1.83 ± 1.21	−4.17 ± 2.67	−2.89 ± 2.10	0.10 ± 1.84	*F*(2,26) = 20.48 *p* < 0.001 (0.61)	*F*(1.59,41.22) = 43.44, *p* < 0.001 (0.63)	*F*(3.17,41.22) = 5.43, *p* = 0.21 (0.11)

**Table 3:** Mean correlations between Tcom and peak object roll for the left, center and right center of mass locations and the overall correlation for all of the center of mass locations combined for each group. Covariation between load force difference (LF difference) and center of pressure (COP) difference for the left, center and right center of mass locations and the overall correlation for all of the center of mass locations combined for each group. Spearman’s correlation was used to obtain the correlation between Tcom and peak roll, while Pearson’s r correlation coefficient was used for LF difference and COP difference. Fisher’s z transformation was used to obtain the average correlation coefficient r for each center of mass location across all participants in each group.

	Left COM	Center COM	Right COM	Overall
Correlation between Tcom and peak roll				
Controls	−0.60	−0.67	−0.70	−0.62
Right Hemisphere Lesion	−0.59	−0.58	−0.29	−0.50
Left Hemisphere Lesion	−0.68	−0.54	−0.41	−0.55
Covariation between LF difference and COP difference				
Controls	−0.69	−0.77	−0.47	−0.79
Right Hemisphere Lesion	−0.57	−0.81	−0.49	−0.65
Left Hemisphere Lesion	−0.53	−0.82	−0.68	−0.70

## Data Availability

The data presented in this study are available on request from the corresponding author. The data are not publicly available due to privacy of the participants.
